# METTL3 Increases Temozolomide Resistance in MGMT‐Negative Glioblastoma by Enhancing CBX5 mRNA Stability in an m^6^A‐IGF2BP2‐Dependent Manner

**DOI:** 10.1002/cns.71164

**Published:** 2026-10-01

**Authors:** Hui Deng, Ruoyu Chen, Daohong Qu, Cifu Qu, Jia Shi

**Affiliations:** ^1^ Department of Neurosurgery The Third Affiliated Hospital of Soochow University Changzhou China; ^2^ Centre of Clinical Laboratory The First Affiliated Hospital of Soochow University Suzhou China

**Keywords:** CBX5, glioblastoma, IGF2BP2, METTL3, N^6^‐methyladenosine, temozolomide resistance

## Abstract

**Aims:**

We examined whether chromobox protein 5 (CBX5) contributes to temozolomide (TMZ) resistance in O^6^‐methylguanine‐DNA methyltransferase (MGMT)‐negative glioblastoma (GBM) and whether it is regulated by methyltransferase‐like 3 (METTL3)‐dependent N^6^‐methyladenosine (m^6^A) and insulin‐like growth factor 2 mRNA‐binding protein 2 (IGF2BP2).

**Methods:**

Public datasets, clinical specimens, MGMT‐negative cells, and subcutaneous xenografts were examined using functional, mechanistic, and rescue assays.

**Results:**

In the CGGA693 cohort of the Chinese Glioma Genome Atlas, higher CBX5 expression remained associated with shorter survival after multivariable adjustment (hazard ratio 1.178, 95% confidence interval 1.050–1.322; *p* = 0.005). CBX5 depletion increased TMZ sensitivity, apoptosis, and DNA damage, reduced ATM phosphorylation and RAD51 abundance, and restricted TMZ‐treated xenograft growth; overexpression produced reciprocal effects in vitro. METTL3 depletion reduced CBX5 expression, mRNA stability, m^6^A enrichment, and wild‐type reporter activity but did not significantly affect the m^6^A motif‐mutant reporter. IGF2BP2 depletion reduced CBX5 protein and mRNA stability, whereas RNA immunoprecipitation supported an IGF2BP2–CBX5 mRNA association. CBX5 re‐expression partially reversed METTL3 depletion‐induced effects on viability and DNA damage.

**Conclusion:**

METTL3‐dependent m^6^A modification and IGF2BP2‐associated stabilization help maintain CBX5 expression, contributing to TMZ resistance and ATM–RAD51‐associated signaling in MGMT‐negative GBM models.

## Graphical abstract

METTL3‐dependent m^6^A modification and IGF2BP2‐associated CBX5 mRNA stabilization help maintain CBX5 expression in MGMT‐negative glioblastoma. During temozolomide (TMZ) treatment, CBX5 contributes to ATM–RAD51‐associated signaling, limits persistent DNA damage and apoptosis, and thereby supports cell survival and TMZ resistance.

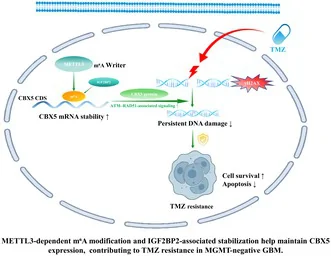

**Abbreviations**
CBX5: chromobox protein 5
CGGA: Chinese Glioma Genome Atlas
DDR: DNA damage response
GBM: glioblastoma
GEPIA: Gene Expression Profiling Interactive Analysis
HR: homologous recombination
IGF2BP2: insulin‐like growth factor 2 mRNA‐binding protein 2
m^6^A: N^6^‐methyladenosine
METTL3: methyltransferase‐like 3
MGMT: O^6^‐methylguanine‐DNA methyltransferase
NHEJ: non‐homologous end joining
SRAMP: Sequence‐based RNA Adenosine Methylation Site Predictor
TCGA: The Cancer Genome Atlas
TMZ: temozolomide
WHO: World Health Organization

## 1 Introduction

Among primary malignant brain tumors in adults, glioblastoma (GBM) is the most frequently diagnosed and remains highly aggressive, with a dismal prognosis [1, 2, 3]. Even after combined‐modality treatment, the reported median survival is approximately 12.1 months, and durable survival remains uncommon [4, 5]. Current clinical management generally comprises the greatest safely achievable surgical resection, followed by radiotherapy with temozolomide (TMZ) administered concurrently and subsequently as adjuvant treatment [6]. Nevertheless, population‐based estimates indicate that the 5‐year relative survival rate across all age groups is only approximately 7% [7]. As an orally administered alkylating agent capable of crossing the blood–brain barrier, TMZ is a central component of first‐line therapy for newly diagnosed GBM; however, the development of resistance markedly diminishes its clinical efficacy [4, 8]. Elucidating the biological processes that enable TMZ resistance may therefore help guide the development of more effective therapeutic approaches [8].

TMZ treatment generates several DNA methyl adducts, including N^7^‐methylguanine, N^3^‐methyladenine, and O^6^‐methylguanine [9, 10]. Of these adducts, O^6^‐methylguanine (O^6^‐MeG) is considered the principal lesion underlying the cytotoxic activity of TMZ [10]. O^6^‐methylguanine‐DNA methyltransferase (MGMT) counteracts this damage by transferring the methyl group from O^6^‐MeG to itself, thereby limiting TMZ‐induced tumor‐cell killing [11]. Hypermethylation of the MGMT promoter reduces MGMT expression and is generally associated with greater TMZ sensitivity, whereas the unmethylated state is commonly linked to higher MGMT abundance and reduced responsiveness to TMZ [12, 13]. MGMT promoter methylation is detected in approximately 40%–50% of GBMs [14]. However, resistance and disease recurrence are also observed in GBM with little or no detectable MGMT, suggesting that additional resistance pathways operate independently of MGMT [9, 15].

Chromobox protein 5 (CBX5), commonly referred to as heterochromatin protein 1α (HP1α), binds di‐ and trimethylated lysine 9 of histone H3 (H3K9me2/3), thereby contributing to heterochromatin organization and transcriptional repression [16]. Previous studies have linked CBX5 to tumor biology in glioma [9, 17, 18], leukemia [19], lung cancer [20, 21], breast cancer [22], intrahepatic cholangiocarcinoma [23], renal cell carcinoma [24], prostate cancer [25, 26], and ovarian cancer [27]. Beyond its role in chromatin organization, CBX5 can accumulate at sites of DNA damage and has been associated with DNA damage‐response signaling and homologous recombination (HR)‐related repair processes [28, 29, 30, 31, 32]. An increase in chromatin‐associated CBX5 following TMZ exposure has also been observed in glioma cells [33]. Despite these observations, whether CBX5 functionally influences TMZ responsiveness in GBM, how it relates to the persistence of TMZ‐induced DNA damage, and which upstream mechanisms control its expression have not been fully defined.

In this study, we first assessed the clinical associations of CBX5 and then determined whether altering its expression influenced the response of MGMT‐negative GBM models to TMZ. Functional analyses evaluated cell survival, apoptotic responses, the persistence of TMZ‐induced DNA damage, ATM–RAD51‐associated signaling, and selected markers related to non‐homologous end joining (NHEJ). To investigate the upstream regulation of CBX5 messenger RNA (mRNA), we examined the involvement of methyltransferase‐like 3 (METTL3)‐dependent N^6^‐methyladenosine (m^6^A) modification and insulin‐like growth factor 2 mRNA‐binding protein 2 (IGF2BP2)‐associated transcript stabilization. Rescue experiments were further performed to determine whether restoration of CBX5 could counteract the effects of METTL3 depletion during TMZ treatment. Through these analyses, we sought to define the regulation of CBX5 and its functional contribution to TMZ resistance in MGMT‐negative GBM.

## 2 Materials and Methods

### 2.1 Cells and Human Tissue Samples

The U87, T98G, SNB19, and LN229 human glioblastoma cell lines were sourced from the American Type Culture Collection (ATCC). Cells were routinely maintained in Dulbecco's Modified Eagle Medium (DMEM; Gibco) containing 10% fetal bovine serum (FBS; Sigma‐Aldrich) and 1% penicillin–streptomycin (Gibco) at 37°C under humidified conditions with 5% CO_2_. Based on Western blot analysis of MGMT protein, U87, SNB19, and LN229 were classified as MGMT‐negative, whereas T98G was classified as MGMT‐positive.

In 2022, paired GBM and adjacent non‐tumor brain specimens were obtained from three patients treated surgically in the Department of Neurosurgery, The Third Affiliated Hospital of Soochow University, and were analyzed retrospectively.

### 2.2 Bioinformatic Analysis

CBX5 expression across different tumor types and its relationships with MGMT and METTL3 expression were examined using the Gene Expression Profiling Interactive Analysis (GEPIA) database, which compiles data from The Cancer Genome Atlas (TCGA) and Genotype‐Tissue Expression (GTEx). Spearman's rank correlation was used to evaluate the gene‐expression associations. Datasets from the Chinese Glioma Genome Atlas (CGGA; http://www.cgga.org.cn/) were interrogated to characterize CBX5 and METTL3 expression in glioma and to compare survival across CBX5 expression strata. The multivariable Cox analysis of CGGA693 included only cases with complete data for all covariates (*n* = 476; 306 deaths). CBX5 expression was evaluated in relation to overall survival in a model containing age, World Health Organization (WHO) grade, isocitrate dehydrogenase (IDH) mutation status, and MGMT promoter methylation status. Before modeling, CBX5 expression was transformed as log_2_ (RSEM +1) and standardized; the hazard ratio for CBX5 was expressed per 1‐SD increase. Candidate m^6^A sites within the CBX5 coding sequence (CDS) were computationally predicted with the Sequence‐based RNA Adenosine Methylation Site Predictor (SRAMP; http://www.cuilab.cn/sramp).

### 2.3 Transfections

Transient gene silencing was performed with siRNAs directed against CBX5, METTL3, or IGF2BP2, with a negative‐control siRNA included as the control. The siRNAs (Sangon Biotech) were introduced into cells in Opti‐MEM using Lipofectamine 3000 reagent (Thermo Fisher Scientific) in accordance with the supplier's instructions. Initial screening comprised three siRNAs against CBX5, two against METTL3, and three against IGF2BP2. On the basis of knockdown efficiency, si‐CBX5‐2, si‐METTL3‐2, and si‐IGF2BP2–2 were carried forward for subsequent experiments. Their sequences were:

si‐CBX5‐2 sense: 5′‐GCCGAUGACAUCAAAUCUAAATT‐3′, antisense: 5′‐UUUAGAUUUGAUGUCAUCGGCTT‐3′.

si‐METTL3‐2 sense: 5′‐CGUCAGUAUCUUGGGCAAGUUTT‐3′, antisense: 5′‐AACUUGCCCAAGAUACUGACGTT‐3′.

si‐IGF2BP2–2 sense: 5′‐CCGUUGUCAACGUCACAUA‐3′, antisense: 5′‐UAUGUGACGUUGACAACGG‐3′.

Stable CBX5 knockdown and overexpression models were generated using lentiviral vectors supplied by GeneChem and encoding a CBX5‐specific shRNA or CBX5 overexpression construct, respectively. At 12–16 h after exposure, the lentiviral inoculum was exchanged for fresh complete culture medium. Stable polyclonal populations were obtained by fluorescence‐based selection and verified 48–72 h after transduction.

For rescue studies, si‐NC or si‐METTL3‐2 was introduced into cells expressing either the control vector or CBX5. The resulting experimental conditions were oe‐NC + si‐NC, oe‐NC + si‐METTL3‐2, and oe‐CBX5 + si‐METTL3‐2.

### 2.4 Real‐Time Quantitative PCR (RT‐qPCR)

Cell lysates were processed with TRIzol, followed by chloroform‐mediated phase separation, and RNA in the aqueous phase was recovered by isopropanol precipitation. The RNA pellets were rinsed with 75% ethanol, allowed to air‐dry, and dissolved in RNase‐free water. RNA yield and purity were determined with a NanoDrop spectrophotometer (Thermo Fisher Scientific). For reverse transcription, 500 ng of total RNA was processed with the NovoScript Plus cDNA Synthesis Kit (Novoprotein). The resulting cDNA was amplified on an ABI 7900HT system (Applied Biosystems) using 2× NovoStart SYBR SuperMix Plus (Novoprotein). Relative transcript abundance was determined by the 2^(−ΔΔCt)^ method, with GAPDH serving as the internal reference gene. The primer sequences are provided in Table S1.

### 2.5 Western Blotting

Protein lysates were prepared from cells or tissues using ice‐cold RIPA buffer containing protease and phosphatase inhibitors. Total protein was quantified with a BCA assay kit (Beyotime Biotechnology). Equivalent protein amounts were resolved by SDS‐PAGE and transferred onto PVDF membranes. The membranes were blocked in 5% non‐fat milk and exposed to primary antibodies at 4°C overnight. After washing, HRP‐conjugated secondary antibodies were applied for 1 h at room temperature. Chemiluminescent signals were developed with an ECL substrate (Meilun Bio), and band densitometry was performed in ImageJ software (NIH). Detailed antibody information is provided in Table S2. For quantitative analysis, p‐ATM and p‐DNA‐PKcs signals were normalized to ATM and DNA‐PKcs, respectively; the Cleaved Caspase‐3/Caspase‐3 and BAX/BCL‐2 ratios were used for apoptosis‐related analyses; γH2AX and the remaining target proteins were normalized to GAPDH.

### 2.6 CCK‐8 Assay

Cells were plated at 3 × 10^3^ cells per well in 96‐well plates. For the Figure 2C dose–response analysis, five independent biological experiments were performed with SNB19 cells and four with LN229 cells. After attachment, cells were exposed to serial TMZ concentrations for 24 h. CCK‐8 reagent (10 μL/well; Beyotime Biotechnology, China) was subsequently added, and the plates were incubated for another 2 h at 37°C. Absorbance was measured at 450 nm using a BioTek microplate reader (USA). Half‐maximal inhibitory concentration (IC_50_) values were estimated by nonlinear regression of the dose–response data in GraphPad Prism 9.5. Because GBM cell lines show considerable heterogeneity and frequently exhibit high TMZ IC_50_ values after short‐term exposure, a broad range of TMZ concentrations was examined to define the dose–response curves more reliably [34].

### 2.7 EdU Assay

Proliferative activity was measured by EdU incorporation with an EdU assay kit (Beyotime Biotechnology, China), following the supplier's protocol. After TMZ exposure, cells received 20 μM EdU for 2 h. The cells were subsequently fixed and permeabilized before click‐reaction labeling, and the EdU‐positive fraction was determined by flow cytometry.

### 2.8 Apoptosis Assay

After TMZ exposure, cells were collected and rinsed with phosphate‐buffered saline. Annexin V‐FITC/PI labeling was then performed with an apoptosis detection kit (Beyotime Biotechnology, China) in accordance with the supplier's protocol, and the proportion of apoptotic cells was quantified by flow cytometry.

### 2.9 TUNEL Staining

DNA fragmentation associated with late‐stage apoptosis was evaluated with a TUNEL assay kit (Beyotime Biotechnology, China). After fixation and permeabilization, cells were incubated with the TUNEL reaction mixture for 1 h at 37°C while protected from light. The TUNEL‐positive fraction was determined by flow cytometry.

### 2.10 Immunofluorescence Staining

Fixed cells grown on coverslips were treated with 0.5% Triton X‐100 for permeabilization and blocked with 5% BSA. Samples were incubated at 4°C overnight with an anti‐γH2AX antibody (1:200; Cell Signaling Technology, USA), followed by an Alexa Fluor 488‐conjugated secondary antibody (1:500; Cell Signaling Technology, USA) for 1 h at room temperature. After nuclear counterstaining with DAPI, images were recorded using a fluorescence microscope (Olympus, Japan). Nuclear γH2AX foci were counted in ImageJ and expressed as the mean number of foci per nucleus.

### 2.11 Alkaline Comet Assay

Alkaline comet analysis of DNA strand breaks was carried out with the CometAssay kit (Trevigen, USA) according to the instructions supplied with the kit. Cells were mixed with low‐melting‐point agarose and mounted on slides before lysis, alkaline DNA unwinding, and electrophoresis. Following staining with SYBR Gold, comet images were acquired by fluorescence microscopy. Tail moment was calculated with CometScore software (TriTek Corp., USA) as the measure of DNA damage. For each experimental group, at least 50 cells were analyzed in each independent experiment.

### 2.12 RNA Stability Assay

To monitor CBX5 mRNA decay, cells transfected with control siRNA or siRNAs targeting METTL3 or IGF2BP2 received 5 μg/mL actinomycin D to block transcription. Samples were harvested from SNB19 cells at 0, 2, 4, and 6 h and from LN229 cells at 0, 1, 2, and 3 h. CBX5 mRNA abundance at each time point was measured by RT‐qPCR and expressed relative to the corresponding 0‐h baseline.

### 2.13 RNA Immunoprecipitation (RIP)‐qPCR

RIP‐qPCR was performed using lysates prepared from SNB19 and LN229 cells. Each lysate was incubated overnight at 4°C with either an anti‐IGF2BP2 antibody or an equivalent amount of normal IgG. Protein A/G magnetic beads were used to recover the antibody‐associated RNA–protein complexes. Following bead washing and protein digestion, the immunoprecipitated RNA was isolated and subjected to RT‐qPCR to quantify CBX5 mRNA. Fold enrichment was calculated relative to the IgG control.

### 2.14 Methylated RNA Immunoprecipitation (MeRIP)

MeRIP was performed as follows. Fragmented RNA was combined with an anti‐m^6^A antibody or normal IgG and incubated overnight at 4°C. Protein A/G magnetic beads were then used to recover the resulting immunocomplexes. Following thorough washing, the bead‐bound RNA was eluted, purified, and subjected to qPCR to evaluate m^6^A enrichment.

### 2.15 Dual‐Luciferase Reporter Assay

Reporter plasmids were generated by inserting CBX5 coding sequence fragments containing either the wild‐type putative m^6^A sites or the corresponding mutated sequences into a dual‐luciferase reporter vector. SNB19 and LN229 cells were co‐transfected with these reporter constructs together with control or METTL3‐targeting siRNA. At 48 h after transfection, firefly and Renilla luciferase signals were determined with a dual‐luciferase reporter assay system. Reporter activity was expressed as the ratio of firefly to Renilla luciferase activity.

### 2.16 Colony Formation Assay

For clonogenic growth analysis, 5 × 10^3^ cells were plated in each well of a 6‐well plate. Following TMZ exposure, cells were maintained in fresh complete medium, which was renewed at intervals of 3–4 days. At the end of the 14‐day culture period, colonies were fixed in ethanol, stained with 0.1% crystal violet (Macklin, China), and photographed. The area occupied by colonies was measured with ImageJ software.

### 2.17 Subcutaneous Xenograft Tumor Model

Female BALB/c nude mice aged 4–6 weeks were randomly assigned to four groups (*n* = 6 per group). According to group assignment, each mouse was subcutaneously inoculated in the right flank with 5 × 10^6^ SNB19‐sh‐NC, SNB19‐sh‐CBX5, LN229‐sh‐NC, or LN229‐sh‐CBX5 cells. Once tumor volume reached approximately 100 mm^3^, TMZ was administered to all animals by oral gavage at 100 mg/kg/day, 5 days per week, for three cycles. Tumor length and width were recorded regularly with calipers and used to calculate tumor volume according to the following formula: (length × width^2^)/2. On day 30, the mice were euthanized, after which tumors were removed, weighed, and prepared for subsequent analyses. Investigators responsible for tumor measurements and histopathological quantification remained blinded to the group assignments.

### 2.18 H&E and Immunohistochemistry (IHC) Staining

Paraffin‐embedded tumor samples were sectioned at 4 μm. Sections intended for histopathological examination were stained with hematoxylin and eosin (H&E) according to standard procedures. For IHC analysis, antigen retrieval and peroxidase blocking were completed before overnight incubation at 4°C with primary antibodies targeting CBX5, Ki67, Cleaved Caspase‐3, and γH2AX. HRP‐conjugated secondary antibodies were subsequently applied. DAB was used for chromogenic development, followed by hematoxylin counterstaining. Stained sections were examined with a light microscope (Olympus, Japan). ImageJ was used to measure the percentage of positively stained area across five randomly selected fields from each tumor.

### 2.19 Statistical Analysis

Unless otherwise indicated, quantitative data are reported as the mean ± SEM from a minimum of three independent biological experiments. Statistical analyses were conducted with GraphPad Prism 9.5 and R software, as applicable. Two‐group comparisons were evaluated using two‐tailed paired or unpaired Student's *t*‐tests, selected according to the experimental design. For multiple comparisons, one‐ or two‐way ANOVA with Dunnett's, Tukey's, or Šídák's multiple‐comparisons test, or multiple unpaired *t*‐tests with false discovery rate (FDR) correction, was used as specified in the figure legends. Longitudinal tumor growth data were evaluated by two‐way repeated‐measures ANOVA. IC_50_ values were derived from dose–response curves fitted by nonlinear regression. Kaplan–Meier survival distributions were assessed with two‐sided log‐rank tests, whereas hazard ratios and their 95% confidence intervals were estimated by multivariable Cox regression. All tests were two‐sided, with *p* < 0.05 considered significant unless otherwise specified.

## 3 Results

### 3.1 CBX5 Is Upregulated in GBM and Associated With Unfavorable Clinical Outcomes

We initially investigated the expression of CBX5 in relation to the clinical characteristics of glioma. Analysis of the GEPIA dataset indicated elevated CBX5 expression in glioblastoma (GBM), low‐grade glioma (LGG), cholangiocarcinoma (CHOL), diffuse large B‐cell lymphoma (DLBC), esophageal carcinoma (ESCA), pancreatic adenocarcinoma (PAAD), thymoma (THYM), and uterine carcinosarcoma (UCS) when compared to their corresponding normal tissues (Figure 1A). In alignment with this expression pattern, Western blot analysis of three matched tissue pairs revealed higher CBX5 protein levels in GBM specimens than in adjacent non‐tumor brain samples (Figure 1B).

**FIGURE 1:**
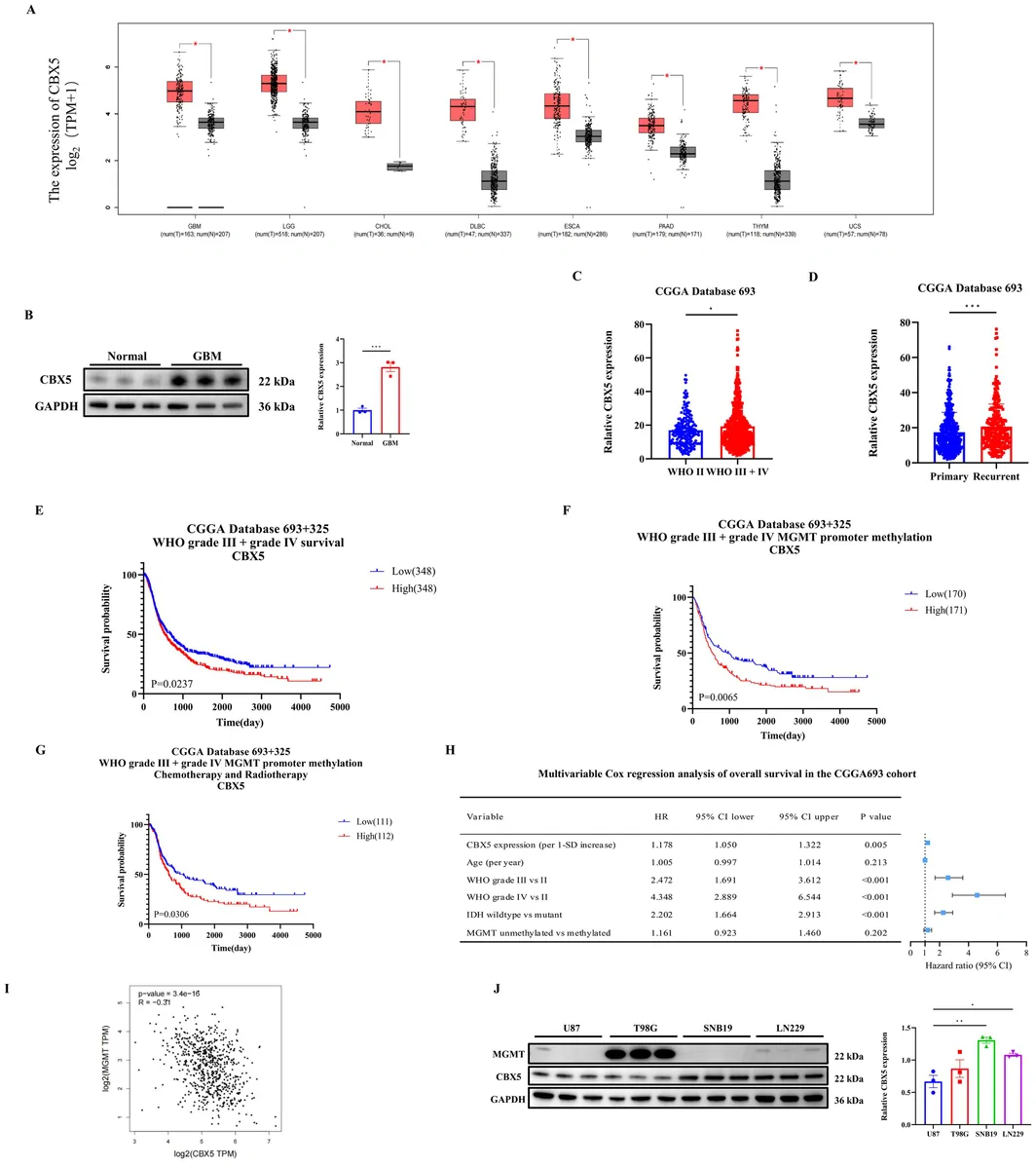
CBX5 is upregulated in GBM and associated with unfavorable clinical outcomes. (A) CBX5 expression analysis across various malignancies from the GEPIA database. Red and gray boxes indicate tumor and normal tissues, respectively. Sample sizes are shown in the plots. CHOL, cholangiocarcinoma; DLBC, diffuse large B‐cell lymphoma; ESCA, esophageal carcinoma; GBM, glioblastoma; LGG, low‐grade glioma; PAAD, pancreatic adenocarcinoma; THYM, thymoma; UCS, uterine carcinosarcoma. (B) Representative Western blots and densitometric quantification of CBX5 protein levels in clinical GBM specimens and matched adjacent non‐tumor brain tissues (*n* = 3 matched pairs). (C) CBX5 expression levels in WHO Grade II (*n* = 188) versus III/IV gliomas (*n* = 504) in the CGGA693 cohort. (D) CBX5 expression in primary (*n* = 422) versus recurrent gliomas (*n* = 271) in the CGGA693 cohort. (E) Overall survival of WHO Grade III/IV glioma patients stratified by CBX5 expression in the combined CGGA693 and CGGA325 cohorts (low, *n* = 348; high, *n* = 348). (F) Overall survival of MGMT promoter‐methylated patients with WHO Grade III/IV glioma stratified by CBX5 expression (low, *n* = 170; high, *n* = 171). (G) Overall survival of MGMT promoter‐methylated patients who received both radiotherapy and chemotherapy, stratified by CBX5 expression (low, *n* = 111; high, *n* = 112). (H) Forest plot of the multivariable Cox regression analysis for overall survival in the CGGA693 cohort (*n* = 476; 306 deaths). The model was adjusted for age, WHO grade, IDH mutation status, and MGMT promoter methylation status. CBX5 expression was modeled per 1‐SD increase in log_2_ (RSEM +1)‐transformed expression. Hazard ratios are presented with 95% confidence intervals. (I) Correlation between CBX5 and MGMT mRNA expression in the GEPIA database, determined by Spearman's analysis (*n* = 681). (J) MGMT and CBX5 protein expression in the indicated GBM cell lines was assessed by Western blot, and CBX5 abundance was quantified by densitometric analysis. Data are presented as the mean ± SEM where applicable. Statistical significance was assessed using a two‐tailed paired Student's *t*‐test in (B), two‐tailed unpaired Student's *t*‐tests in (C) and (D), two‐sided log‐rank tests in (E)–(G), multivariable Cox regression in (H), Spearman's correlation analysis in (I), and one‐way ANOVA followed by Tukey's multiple‐comparisons test in (J). *p < 0.05, **p < 0.01, ***p < 0.001.

In the CGGA693 cohort, CBX5 expression was significantly higher in WHO Grade III/IV gliomas compared to Grade II gliomas, and in recurrent tumors relative to primary tumors (Figure 1C,D). An analysis of the combined CGGA693 and CGGA325 cohorts revealed that patients with WHO Grade III/IV glioma in the high‐CBX5 group experienced shorter overall survival (Figure 1E). This adverse survival association was also evident in tumors with MGMT promoter methylation (Figure 1F), including those treated with both radiotherapy and chemotherapy (Figure 1G). The complete‐case multivariable analysis of CGGA693 encompassed 476 patients and recorded 306 deaths. Each 1‐SD increase in CBX5 expression was associated with an elevated hazard of death (hazard ratio = 1.178, 95% CI = 1.050–1.322, *p* = 0.005) after adjusting for age, WHO grade, IDH mutation status, and MGMT promoter methylation status as covariates (Figure 1H). Therefore, higher CBX5 expression was associated with unfavorable clinicopathological features and outcomes in the glioma cohorts studied. Furthermore, GEPIA analysis identified an inverse Spearman correlation between CBX5 and MGMT expression (Figure 1I). Among the GBM cell lines evaluated, SNB19 and LN229 exhibited undetectable MGMT protein levels while displaying relatively high CBX5 expression, leading to their selection for subsequent experiments (Figure 1J).

### 3.2 CBX5 Knockdown Sensitizes MGMT‐Negative GBM Cells to TMZ

To assess the role of CBX5 in the response to TMZ, CBX5 was transiently silenced using siRNA in SNB19 and LN229 cells. RT‐qPCR and Western blotting confirmed the reduction of CBX5 at both the transcript and protein levels (Figure 2A,B). Following 24 h of TMZ exposure, silencing of CBX5 shifted the IC_50_ from 791.1 μM to 596.7 μM in SNB19 cells and from 1649 μM to 1124 μM in LN229 cells (Figure 2C). Based on these dose–response profiles, 400 μM TMZ was chosen for SNB19 cells and 1200 μM for LN229 cells in subsequent assays. EdU‐based flow cytometry revealed a reduced proportion of proliferating cells after CBX5 depletion (Figure 2D). Annexin V‐FITC/PI analysis identified an increased apoptotic fraction in CBX5‐silenced cells during TMZ treatment (Figure 2E), a finding corroborated by TUNEL analysis (Figure 2F). Immunoblotting further indicated that 24‐h TMZ exposure elevated the Cleaved Caspase‐3/Caspase‐3 and BAX/BCL‐2 ratios in cells deficient in CBX5 (Figure 2G). These coordinated protein alterations are indicative of the activation of the intrinsic mitochondrial apoptotic pathway [35]. Collectively, these findings demonstrate that depletion of CBX5 sensitizes MGMT‐negative GBM cells to TMZ.

**FIGURE 2:**
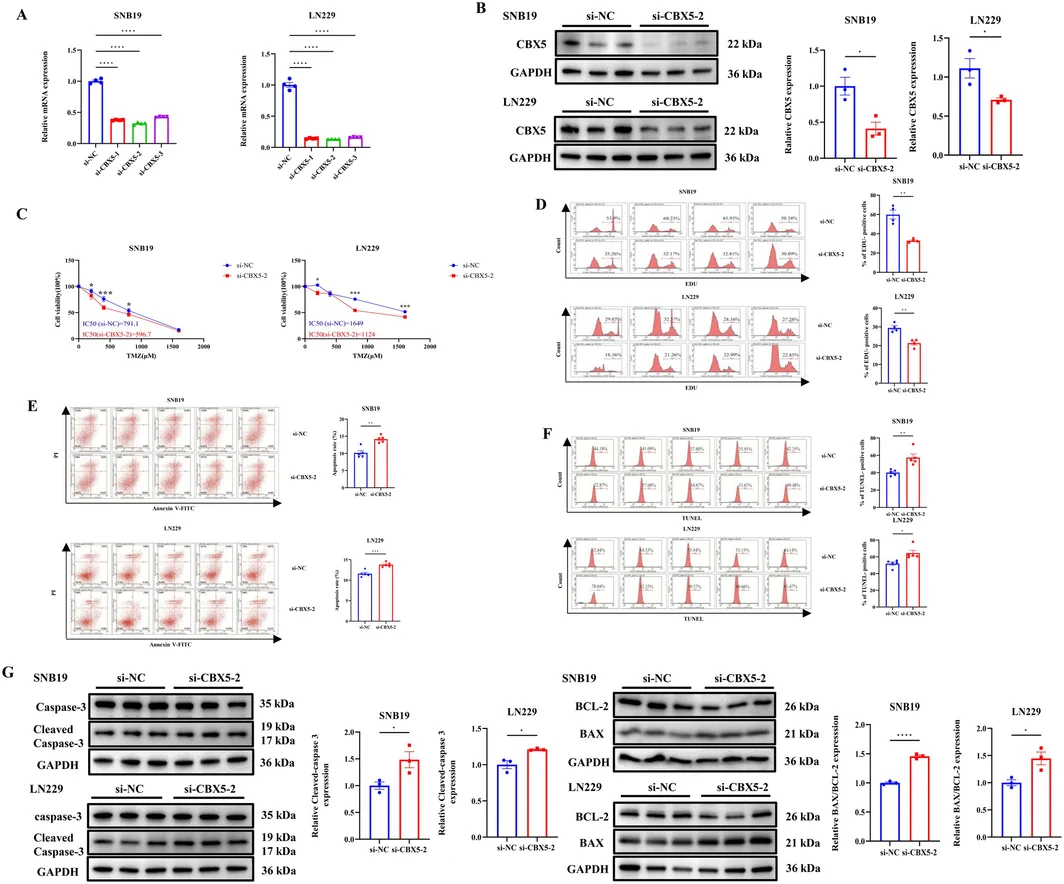
CBX5 knockdown sensitizes MGMT‐negative GBM cells to TMZ. (A) CBX5 mRNA levels after knockdown, assessed by RT‐qPCR in cells transfected with si‐NC or one of three CBX5‐targeting siRNAs (*n* = 4). (B) CBX5 protein expression after knockdown, assessed by Western blot and densitometric analysis. (C) TMZ sensitivity measured by CCK‐8 assays after 24 h treatment. Five and four independent biological experiments were performed for SNB19 and LN229 cells, respectively. IC_50_ values are shown and were estimated by nonlinear regression. (D) Cell proliferation analyzed by EdU incorporation flow cytometry after TMZ treatment (*n* = 4). (E) Apoptosis rates determined by Annexin V‐FITC/PI flow cytometry following TMZ treatment (*n* = 5). (F) Apoptotic cells detected by TUNEL staining and quantified by flow cytometry (*n* = 5). (G) Expression of Caspase‐3, Cleaved Caspase‐3, BAX, and BCL‐2 after TMZ treatment, assessed by Western blot. The Cleaved Caspase‐3/Caspase‐3 and BAX/BCL‐2 ratios were quantified. Data are presented as the mean ± SEM. Statistical significance was assessed using one‐way ANOVA followed by Dunnett's multiple‐comparisons test in (A), two‐way ANOVA followed by Šídák's multiple‐comparisons test in (C), and two‐tailed unpaired Student's *t*‐tests in (B) and (D)–(G). *p < 0.05, **p < 0.01, ***p < 0.001, ****p < 0.0001.

### 3.3 CBX5 Modulates TMZ‐Induced DNA Damage and ATM–RAD51‐Associated Signaling

To evaluate the impact of CBX5 on the persistence of TMZ‐induced DNA lesions, we analyzed γH2AX, a well‐established marker for DNA double‐strand break signaling. Western blotting revealed a greater accumulation of γH2AX in CBX5‐depleted cells subjected to TMZ treatment (Figure 3A). Additionally, immunofluorescence analysis demonstrated a corresponding increase in nuclear γH2AX foci (Figure 3B). Alkaline comet analysis further corroborated these findings, showing an elevated tail moment following CBX5 knockdown (Figure 3C), which indicates a heightened residual burden of DNA strand breaks after TMZ exposure. Collectively, these results suggest that the loss of CBX5 enhances the persistence of TMZ‐induced DNA damage, potentially contributing to an improved drug response.

**FIGURE 3:**
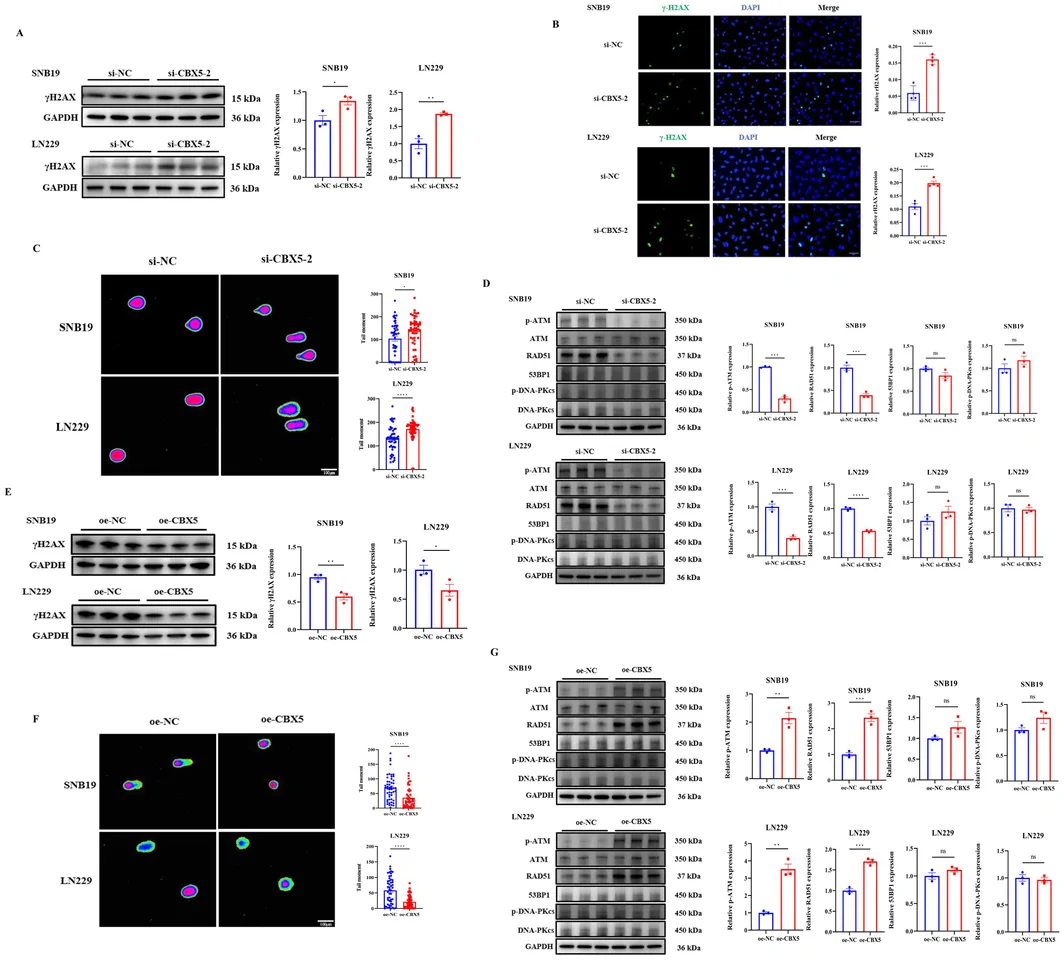
CBX5 modulates TMZ‐induced DNA damage and ATM–RAD51‐associated signaling. (A) γH2AX protein levels after TMZ treatment, assessed by Western blot and densitometric analysis. (B) Representative immunofluorescence images and quantification of γH2AX foci (green). Nuclei were counterstained with DAPI (blue). Scale bar, 20 μm. (C) Representative alkaline comet assay images and tail moment quantification. At least 50 cells per group were analyzed in each independent experiment. Scale bar, 100 μm. (D) Western blot and densitometric analyses of p‐ATM/ATM, RAD51, 53BP1, and p‐DNA‐PKcs/DNA‐PKcs in CBX5‐knockdown cells following TMZ treatment. (E) γH2AX protein levels in control and CBX5‐overexpressing cells following TMZ treatment, assessed by Western blot and densitometric analysis. (F) Representative alkaline comet assay images and tail moment quantification in control and CBX5‐overexpressing cells. At least 50 cells per group were analyzed in each independent experiment. Scale bar, 100 μm. (G) Western blot and densitometric analyses of p‐ATM/ATM, RAD51, 53BP1, and p‐DNA‐PKcs/DNA‐PKcs in control and CBX5‐overexpressing cells following TMZ treatment. Phosphorylated ATM and DNA‐PKcs were normalized to the corresponding total proteins, whereas RAD51 and 53BP1 were normalized to GAPDH. Data are presented as the mean ± SEM. Statistical comparisons were performed using two‐tailed unpaired Student's *t*‐tests. ns, not significant. *p < 0.05, **p < 0.01, ***p < 0.001, and ****p < 0.0001.

To obtain reciprocal evidence, we investigated DNA damage following the overexpression of CBX5, which was confirmed at both the mRNA and protein levels (Figure S1A,B). The overexpression of CBX5 led to a reduction in γH2AX levels and a decrease in comet tail moments after TMZ treatment (Figure 3E,F). Additionally, CBX5 overexpression enhanced cell viability and clonogenic survival while mitigating changes in pro‐apoptotic protein levels under TMZ treatment (Figure S1C–E). Collectively, the results from the knockdown and overexpression experiments demonstrate that the abundance of CBX5 inversely affects the persistence of DNA damage induced by TMZ.

To further elucidate the DNA damage response pathways linked to CBX5, we assessed ATM and DNA‐PKcs phosphorylation and RAD51 and 53BP1 abundance. Following TMZ treatment, knockdown of CBX5 resulted in a decreased p‐ATM/ATM ratio and reduced RAD51 levels in both SNB19 and LN229 cells (Figure 3D), while overexpression of CBX5 yielded the opposite effects (Figure 3G). In contrast, neither the abundance of 53BP1 nor the p‐DNA‐PKcs/DNA‐PKcs ratio exhibited significant changes due to CBX5 knockdown or overexpression. These findings implicate CBX5 in ATM–RAD51‐related DNA damage signaling but do not support a detectable regulation of the assessed NHEJ‐associated markers.

### 3.4 CBX5 Knockdown Suppresses Xenograft Growth During TMZ Treatment

To extend the cell‐based findings in vivo, we generated subcutaneous tumors in nude mice using SNB19 and LN229 cells that stably expressed sh‐NC or sh‐CBX5, with all groups subsequently receiving TMZ. In comparison to sh‐NC tumors, sh‐CBX5 tumors exhibited reduced tumor volumes and endpoint weights in both the SNB19 and LN229 models (Figure 4A–C). Immunohistochemical analysis confirmed diminished CBX5 staining in sh‐CBX5 tumors and revealed decreased Ki67 positivity, along with increased positivity for Cleaved Caspase‐3 and γH2AX, relative to sh‐NC tumors (Figure 4D). These in vivo findings indicate that CBX5 depletion limits GBM xenograft growth during TMZ treatment, which is associated with reduced proliferation, increased apoptosis, and heightened DNA damage.

**FIGURE 4:**
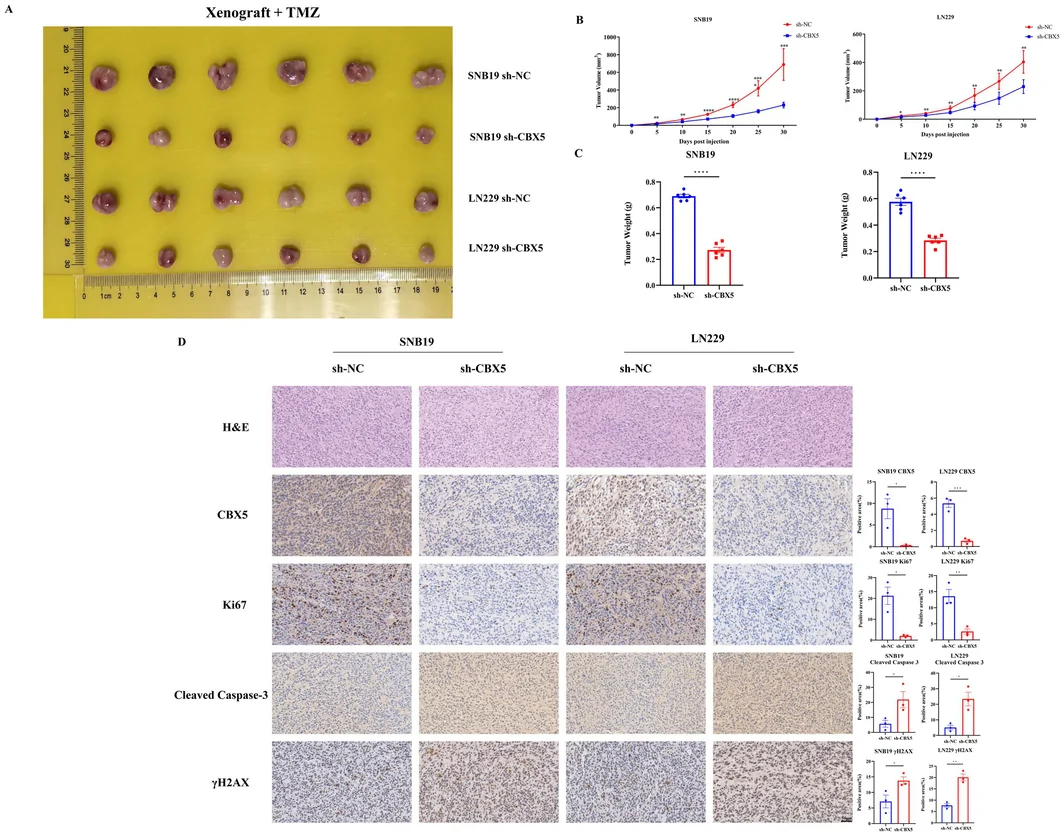
CBX5 knockdown suppresses GBM xenograft growth during TMZ treatment. Female BALB/c nude mice bearing SNB19 or LN229 xenografts stably expressing sh‐NC or sh‐CBX5 received TMZ at 100 mg/kg/day by oral gavage, 5 days per week, for three cycles (*n* = 6 mice per group). (A) Representative images of subcutaneous xenografts at the experimental endpoint. (B) Tumor growth curves during the treatment period. (C) Final tumor weights at the endpoint. (D) H&E staining and immunohistochemical analysis of CBX5, Ki67, Cleaved Caspase‐3, and γH2AX in xenograft sections (*n* = 3). The positively stained area was quantified from five randomly selected fields per tumor. Scale bar, 50 μm. Data are presented as the mean ± SEM. Tumor growth curves were analyzed using two‐way repeated‐measures ANOVA followed by Šídák's multiple‐comparisons test, whereas endpoint tumor weights and IHC quantification were analyzed using two‐tailed unpaired Student's *t*‐tests. *p < 0.05, **p < 0.01, ***p < 0.001, ****p < 0.0001.

### 3.5 METTL3 Regulates CBX5 mRNA Stability Through m^6^A Modification

Analysis of CGGA RNA‐sequencing data showed higher METTL3 expression in glioma samples from the CGGA693 and CGGA325 cohorts than in non‐glioma brain tissues from the CGGA mRNAseq_20 dataset (Figure S2A). We knocked down METTL3 in SNB19 and LN229 cells using two independent siRNAs, both of which reduced METTL3 mRNA expression; si‐METTL3‐2 was selected for subsequent experiments because it produced the greater knockdown efficiency (Figure 5A). Screening of key DNA repair genes revealed that METTL3 knockdown reduced CBX5 mRNA expression in both SNB19 and LN229 cells, although several other transcripts exhibited cell‐line‐dependent changes (Figure S2B). Western blot analysis further showed that si‐METTL3‐2 reduced both METTL3 and CBX5 protein abundance (Figure 5B). Furthermore, actinomycin D chase assays demonstrated that METTL3 knockdown significantly reduced CBX5 mRNA stability (Figure 5C), which was accompanied by reduced m^6^A enrichment on CBX5 mRNA, as determined by MeRIP‐qPCR (Figure 5D). Spearman analysis of the GEPIA data identified a positive association between CBX5 and METTL3 transcript abundance (Figure S2C). Prediction using the SRAMP online tool identified three high‐probability m^6^A sites within the CBX5 CDS (Figure 5E). Luciferase measurements showed that METTL3 depletion significantly lowered reporter activity from the wild‐type CBX5 construct, whereas activity of the construct carrying mutations in the predicted m^6^A motifs was not significantly altered (Figure 5F). Taken together, these results support a role for METTL3‐dependent m^6^A modification in maintaining CBX5 transcript stability and protein abundance.

**FIGURE 5:**
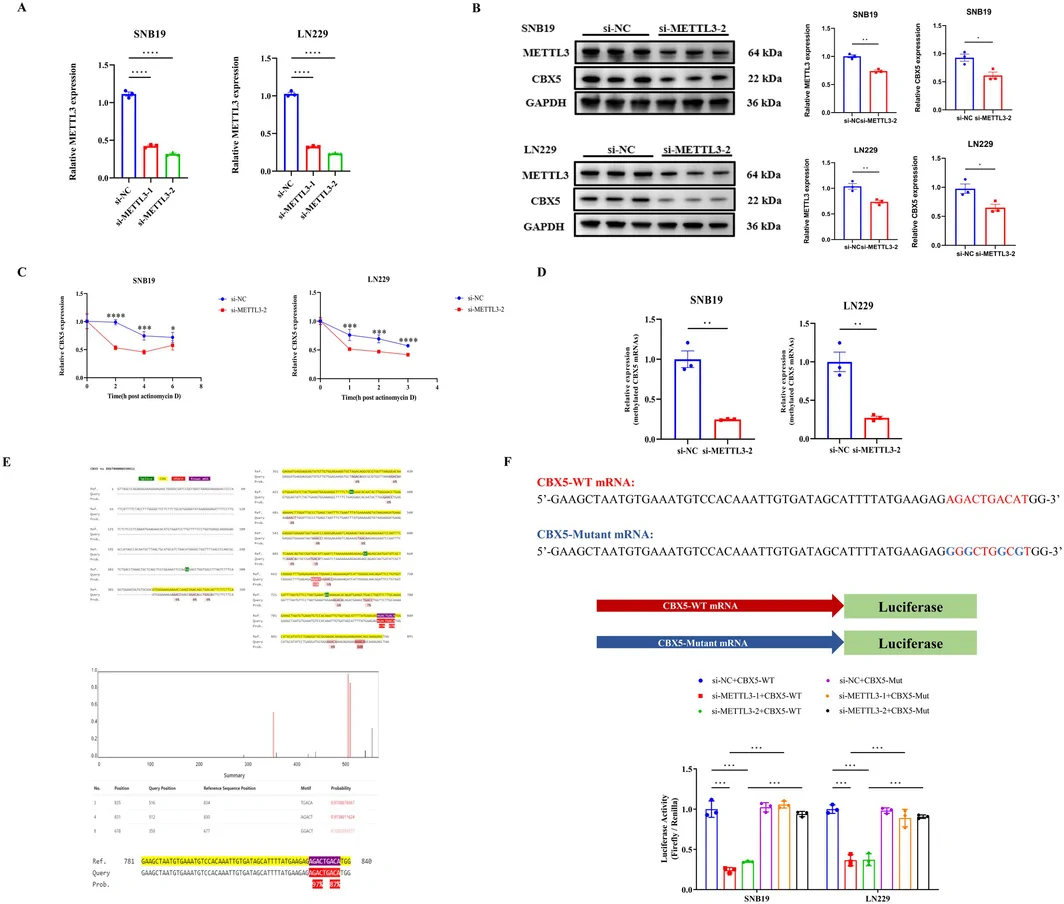
METTL3 regulates CBX5 mRNA stability through m^6^A modification. (A) METTL3 knockdown efficiency in SNB19 and LN229 cells transfected with si‐NC, si‐METTL3‐1, or si‐METTL3‐2, assessed by RT‐qPCR (*n* = 4). (B) Representative Western blots and densitometric quantification of METTL3 and CBX5 protein abundance following transfection with si‐NC or si‐METTL3‐2. (C) CBX5 mRNA decay following treatment with actinomycin D (5 μg/mL) in si‐NC‐ and si‐METTL3‐2‐transfected cells. SNB19 cells were analyzed at 0, 2, 4, and 6 h, and LN229 cells were analyzed at 0, 1, 2, and 3 h. CBX5 mRNA levels were normalized to the corresponding 0‐h value. (D) m^6^A enrichment on CBX5 mRNA following METTL3 knockdown, measured by MeRIP‐qPCR. Enrichment was normalized to total input RNA. (E) SRAMP prediction of three high‐probability m^6^A modification sites within the CBX5 coding sequence. (F) Schematic representation of the wild‐type and m^6^A motif‐mutant CBX5 reporter constructs and corresponding dual‐luciferase reporter activity following co‐transfection with si‐NC, si‐METTL3‐1, or si‐METTL3‐2. Firefly luciferase activity was normalized to Renilla luciferase activity. Data are presented as the mean ± SEM. Statistical significance was assessed using one‐way ANOVA followed by Dunnett‘s multiple‐comparisons test in (A), two‐tailed unpaired Student‘s *t*‐tests in (B) and (D), and two‐way ANOVA followed by Šídák‘s multiple‐comparisons test in (C) and (F). *p < 0.05, **p < 0.01, ***p < 0.001, ****p < 0.0001.

### 3.6 IGF2BP2 Contributes to CBX5 mRNA Stabilization, and CBX5 Partially Mediates the Effects of METTL3 Depletion

To identify a candidate m^6^A reader involved in the regulation of CBX5, we initially compared the basal mRNA expression levels of IGF2BP1/2/3 and YTHDF1/2/3 in SNB19 and LN229 cells. Among these candidates, IGF2BP2 exhibited relatively high expression in both SNB19 and LN229 cells, leading to its selection for further functional testing (Figure 6A). We evaluated three independent siRNAs targeting IGF2BP2, and selected si‐IGF2BP2‐2, which demonstrated the highest knockdown efficiency, for subsequent experiments (Figure 6B). The knockdown of IGF2BP2 resulted in a decrease in CBX5 protein levels in both SNB19 and LN229 cells (Figure 6C). RNA immunoprecipitation followed by qPCR revealed significant enrichment of CBX5 mRNA in the IGF2BP2 immunoprecipitate compared to the IgG control, indicating an association between IGF2BP2 and CBX5 mRNA (Figure 6D). Additionally, actinomycin D chase assays demonstrated accelerated decay of CBX5 mRNA following IGF2BP2 knockdown (Figure 6E). Together with the METTL3‐ and m^6^A motif‐associated results presented in Figure 5, these findings support a role for IGF2BP2 in CBX5 mRNA stabilization within the METTL3/m^6^A‐associated regulatory framework.

**FIGURE 6:**
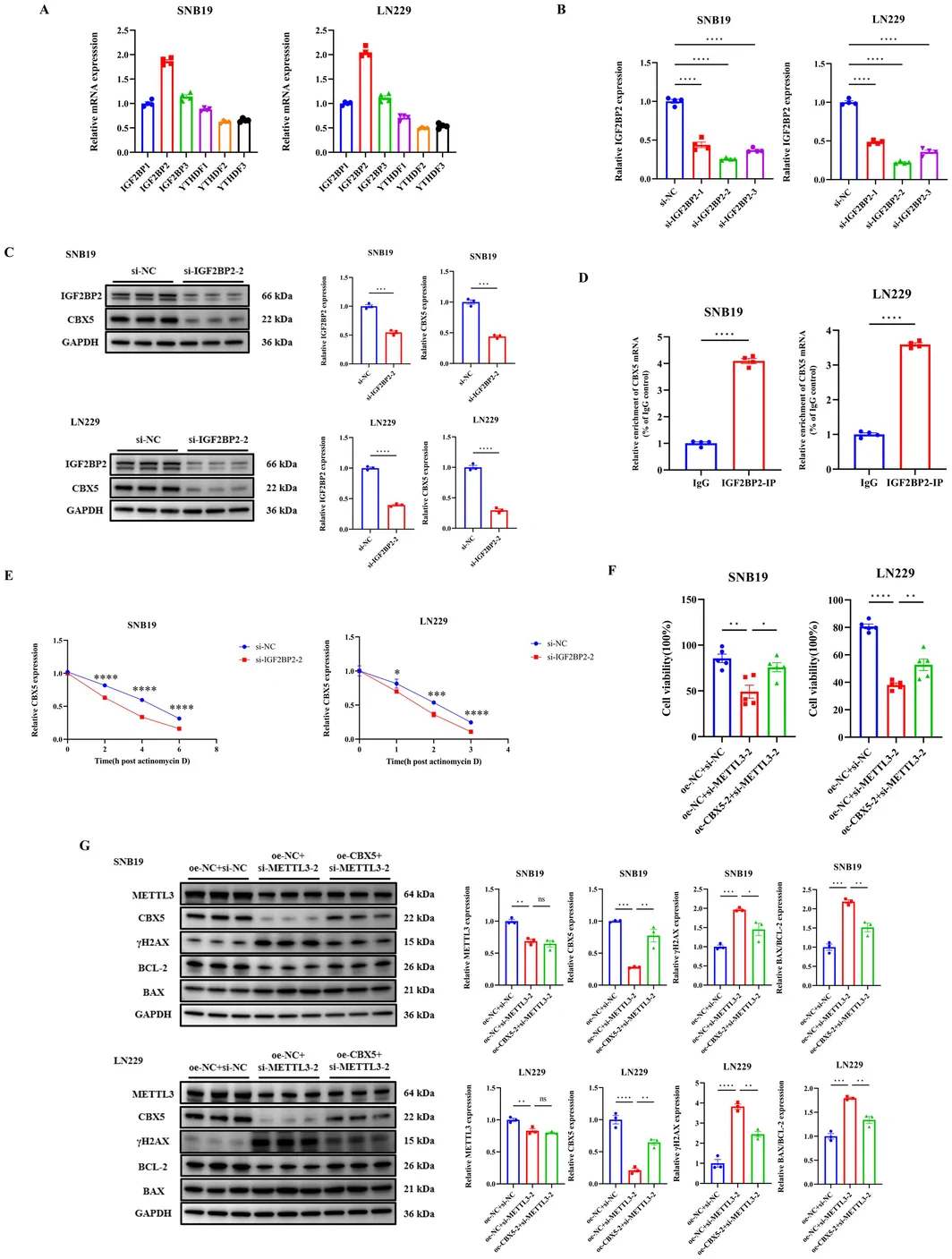
IGF2BP2 contributes to CBX5 mRNA stabilization, and CBX5 re‐expression partially reverses the effects of METTL3 depletion. (A) Basal mRNA expression of the candidate m^6^A readers IGF2BP1, IGF2BP2, IGF2BP3, YTHDF1, YTHDF2, and YTHDF3 in SNB19 and LN229 cells, assessed by RT‐qPCR (*n* = 4). (B) IGF2BP2 knockdown efficiency in SNB19 and LN229 cells transfected with si‐NC or one of three independent IGF2BP2‐targeting siRNAs, assessed by RT‐qPCR (*n* = 4). (C) Representative Western blots and densitometric quantification of IGF2BP2 and CBX5 protein abundance following transfection with si‐NC or si‐IGF2BP2‐2. (D) Enrichment of CBX5 mRNA in IGF2BP2 immunoprecipitates compared with the IgG control, determined by RIP‐qPCR. Results are expressed as fold enrichment relative to the IgG control. (E) CBX5 mRNA decay following actinomycin D treatment in si‐NC‐ and si‐IGF2BP2–2‐transfected cells. SNB19 cells were analyzed at 0, 2, 4, and 6 h, and LN229 cells were analyzed at 0, 1, 2, and 3 h. CBX5 mRNA levels were normalized to the corresponding 0‐h value. Three and four independent biological experiments were performed for SNB19 and LN229 cells, respectively. (F) Cell viability during TMZ treatment in cells transfected with oe‐NC + si‐NC, oe‐NC + si‐METTL3‐2, or oe‐CBX5 + si‐METTL3‐2, assessed using the CCK‐8 assay (*n* = 5). (G) Representative Western blots and densitometric quantification of METTL3, CBX5, γH2AX, BCL‐2, and BAX in the indicated rescue groups. METTL3, CBX5, and γH2AX were normalized to GAPDH, and apoptosis‐related changes were evaluated using the BAX/BCL‐2 ratio. Data are presented as the mean ± SEM. Statistical significance was assessed using one‐way ANOVA followed by Tukey's multiple‐comparisons test in (A), (F), and (G), Dunnett's multiple‐comparisons test in (B), two‐tailed unpaired Student's *t*‐tests in (C), a two‐tailed paired Student's *t*‐test in (D), and two‐way ANOVA followed by Šídák's multiple‐comparisons test in (E). ns, not significant. *p < 0.05, **p < 0.01, ***p < 0.001, and ****p < 0.0001.

We subsequently conducted rescue experiments to ascertain whether CBX5 operates downstream of METTL3. Knockdown of METTL3 resulted in decreased cell viability during TMZ treatment; however, re‐expression of CBX5 partially restored cell viability in both SNB19 and LN229 cells (Figure 6F). Western blot analysis demonstrated that METTL3 levels remained diminished following CBX5 re‐expression, while the abundance of CBX5 protein was restored (Figure 6G). Additionally, re‐expression of CBX5 partially mitigated the increase in γH2AX levels and the BAX/BCL‐2 ratio induced by METTL3 knockdown (Figure 6G). These results indicate that CBX5 serves as a functional downstream mediator that influences the effects of METTL3 on TMZ‐related DNA damage and cell survival.

## 4 Discussion

Frequent recurrence and the emergence of treatment resistance significantly contribute to the poor clinical outcomes associated with GBM [36, 37]. TMZ remains a cornerstone of GBM chemotherapy due to its ability to cross the blood–brain barrier and its generally manageable safety profile [9]. However, resistance to TMZ substantially diminishes tumor control and promotes subsequent progression and recurrence. Despite extensive research, the molecular mechanisms underlying TMZ resistance have not been fully elucidated [38]. The most well‐characterized mechanism involves MGMT, an enzyme that removes the cytotoxic O^6^‐methylguanine (O^6^‐MeG) lesion by covalently accepting its methyl group onto a cysteine residue, thereby irreversibly inactivating MGMT [39, 40]. The repair of O^6^‐MeG mitigates the formation of DNA mismatches and the subsequent generation of strand breaks, which reduces the toxicity associated with alkylating agents. Nevertheless, resistance and recurrence also occur in GBM with minimal or undetectable MGMT expression, indicating the existence of resistance mechanisms beyond MGMT [9, 15]. Therefore, elucidating these additional pathways is crucial for understanding and potentially overcoming TMZ resistance in MGMT‐negative GBM.

CBX5, also referred to as HP1α, is a fundamental component of heterochromatin that facilitates chromatin condensation and epigenetic gene silencing. Emerging evidence has identified CBX5 as an oncogenic factor [19]. Previous studies have documented the upregulation of CBX5 in glioma, where it promotes cell proliferation and exhibits anti‐apoptotic effects [18]. In a related investigation, Sun et al. demonstrated that increased CBX5 expression counteracted the reductions in GBM cell proliferation and survival resulting from LINC02381 silencing [17]. However, the role of CBX5 in chemotherapeutic resistance in GBM has been inadequately explored. Our findings reveal a functional role for CBX5 in modulating TMZ sensitivity in MGMT‐negative SNB19 and LN229 GBM cells. CBX5 knockdown resulted in reduced TMZ IC_50_ values, suppressed proliferation, and increased apoptosis during TMZ treatment. Conversely, CBX5 overexpression enhanced cell viability and clonogenic survival while mitigating changes in apoptosis‐associated proteins under TMZ treatment. In xenografts treated with TMZ, CBX5 depletion in SNB19‐ and LN229‐derived tumors was linked to reduced growth, diminished Ki67 staining, and increased positivity for Cleaved Caspase‐3 and γH2AX. Collectively, these findings support the notion that CBX5 contributes to TMZ resistance in the MGMT‐negative GBM models examined.

The relatively high absolute TMZ IC_50_ values observed in SNB19 and LN229 cells in our study align with prior reports of intrinsic TMZ resistance in in vitro models of glioblastoma. A comprehensive systematic review has indicated that TMZ IC_50_ values in GBM cell lines typically range from several hundred micromolar to over 1000 micromolar, particularly under short‐term exposure conditions, such as 24 h [34, 41, 42]. Consequently, these absolute IC_50_ values must be interpreted concerning the specific cell models, exposure duration, and assay conditions. In contrast, the directional changes following CBX5 perturbation offer more pertinent evidence regarding altered TMZ sensitivity.

As TMZ exerts its cytotoxicity primarily through DNA damage, the efficiency of the DNA damage response (DDR) and repair machinery is an important determinant of resistance. Recent evidence indicates that multiple DDR pathways can buffer the genotoxic stress induced by radiotherapy and alkylating chemotherapy in glioma, thereby contributing to treatment resistance and providing a rationale for DDR‐targeted combination strategies [43]. Li et al. demonstrated that ALDH1A3–PKM2‐driven metabolic reprogramming promoted XRCC1 K247 lactylation and nuclear translocation in glioblastoma stem cells, thereby enhancing DNA repair and resistance to chemoradiotherapy [44]. Previous studies have implicated CBX5 in DDR signaling [29], linked it to defective DNA repair in high‐grade serous ovarian cancer [27], and shown that CBX5 facilitates BRCA1 recruitment to damaged chromatin to preserve homologous recombination fidelity [32]. Consistent with these findings, CBX5 knockdown increased γH2AX foci and alkaline comet tail moments following TMZ treatment, whereas CBX5 overexpression reduced these DNA damage‐associated readouts. These reciprocal effects indicate an inverse association between CBX5 abundance and the persistence of TMZ‐induced DNA damage. At the pathway level, CBX5 knockdown reduced the p‐ATM/ATM ratio and RAD51 abundance, whereas CBX5 overexpression produced the opposite changes. In contrast, neither 53BP1 abundance nor the p‐DNA‐PKcs/DNA‐PKcs ratio was detectably altered by CBX5 perturbation. This pattern links CBX5 to ATM–RAD51‐associated DNA damage signaling in the examined GBM models but does not establish direct enhancement of homologous recombination or detectable regulation of the examined non‐homologous end‐joining‐associated markers. Overall, these findings place CBX5 within the cellular response to TMZ‐induced DNA lesions and suggest that this role contributes to the resistant phenotype.

The mechanisms that regulate CBX5 localization and abundance remain incompletely understood. In a study of MGMT‐negative GBM, Yi et al. utilized subcellular proteomics to identify DHC2 as a direct interacting partner of CBX5. They linked this interaction to the nuclear localization of CBX5, enhanced repair of TMZ‐induced DNA damage, and drug resistance [9]. While this study elucidated a facet of CBX5 localization, the regulation of CBX5 expression was not fully clarified. Previous research indicated that m^6^A deposition by METTL3 can extend the stability of transcripts associated with TMZ resistance [6, 45], aligning with the growing recognition of m^6^A as a crucial regulator in cancer biology [46]. METTL3 serves as the catalytic component of the m^6^A methyltransferase complex and has been implicated in oncogenic functions across various tumor types [47]; our group has also associated METTL3 with TMZ resistance in glioma [6, 45]. In the current study, depletion of METTL3 resulted in reduced CBX5 protein levels, diminished CBX5 mRNA stability, and decreased m^6^A enrichment on CBX5 transcripts. Furthermore, METTL3 depletion lowered the activity of the wild‐type CBX5 reporter but did not significantly impact the reporter with mutations in the predicted m^6^A motifs, suggesting that these motifs are involved in METTL3‐dependent regulation of CBX5. Collectively, the data support the notion that METTL3‐dependent m^6^A deposition contributes to the maintenance of CBX5 mRNA stability and protein levels.

Reader proteins are crucial in determining how m^6^A marks affect the behavior of modified transcripts. The IGF2BP family of proteins has been shown to recognize m^6^A‐modified transcripts, thereby enhancing their stability and translation [48]. Among the m^6^A reader candidates investigated in this study, IGF2BP2 exhibited relatively high basal expression in both SNB19 and LN229 cells, leading to its selection for functional evaluation. Knockdown of IGF2BP2 resulted in decreased abundance of CBX5 protein and accelerated decay of CBX5 mRNA. Furthermore, RIP‐qPCR revealed an enrichment of CBX5 mRNA in IGF2BP2 immunoprecipitates. These results indicate an association between IGF2BP2 and CBX5 mRNA, suggesting that IGF2BP2 may contribute to the m^6^A‐associated stabilization of CBX5. However, since the dependence of IGF2BP2 binding on METTL3 activity or the predicted m^6^A motifs in CBX5 was not directly assessed, the current data do not definitively establish m^6^A‐dependent binding of IGF2BP2 to CBX5 mRNA.

The rescue experiments elucidated the functional relationship between METTL3 and CBX5. Re‐expression of CBX5 partially restored cell viability and mitigated the increases in γH2AX abundance and the BAX/BCL‐2 ratio that were induced by METTL3 depletion during TMZ treatment. This partial restoration supports the notion that CBX5 acts as a functional downstream mediator contributing to the effects of METTL3, while also indicating that other METTL3‐regulated targets may play a role in TMZ resistance. Collectively, the rescue and RNA‐regulatory findings endorse a model in which METTL3‐dependent m^6^A modification and IGF2BP2‐associated transcript stabilization maintain CBX5 expression and contribute to the TMZ‐resistant phenotype observed in MGMT‐negative GBM cells.

From a clinical perspective, elevated expression of CBX5 was correlated with a higher WHO grade, recurrent disease rather than primary disease, and reduced overall survival in the glioma cohorts analyzed. In the multivariable CGGA693 analysis, the relationship between CBX5 expression and shorter survival remained significant after adjusting for age, WHO grade, IDH mutation status, and MGMT promoter methylation status. The adverse outcomes observed in patients with high CBX5 expression within the MGMT promoter‐methylated subgroup further underscore the need to investigate CBX5‐associated resistance mechanisms in this population. However, these findings should not be construed as universal validation of CBX5 as an independent prognostic factor or as evidence that CBX5 predicts response to TMZ. At the functional level, the current data indicate that disrupting METTL3‐dependent m^6^A regulation, IGF2BP2‐associated stabilization of CBX5, or the function of CBX5 could impair the ability of MGMT‐negative GBM cells to endure TMZ‐induced DNA damage. Given that inhibitor‐based experiments, combination treatments, and drug‐synergy analyses were not performed, this potential therapeutic implication necessitates thorough preclinical evaluation.

Several limitations should be acknowledged. First, our in vivo evidence was obtained using subcutaneous xenografts established in immunodeficient mice. This experimentally tractable model provides a practical and reproducible means of monitoring tumor growth and treatment response and has been used in representative studies of TMZ resistance and therapeutic sensitization in GBM [40, 49]. Nevertheless, it does not fully reproduce the intracranial microenvironment, blood–brain barrier, infiltrative growth pattern, or intact immune context. Further evaluation in orthotopic intracranial models could provide complementary information regarding these aspects and represents an important direction for future work. Second, protein‐level validation in clinical tissues was limited to three paired GBM specimens, whereas the survival analyses relied mainly on retrospective public datasets encompassing heterogeneous glioma grades, molecular subtypes, and treatment backgrounds. Although CBX5 showed an adjusted prognostic association in the CGGA693 cohort, validation in additional independent and molecularly annotated GBM cohorts, particularly cohorts with detailed TMZ treatment and response data, would help clarify its clinical relevance. Third, the changes in DDR‐associated proteins represent pathway‐related readouts rather than direct functional measurements of homologous recombination or non‐homologous end joining, and the dependence of IGF2BP2 binding on METTL3 activity or the predicted CBX5 m^6^A motifs was not directly examined. Future studies integrating pathway‐specific repair analyses, m^6^A site‐dependent binding experiments, and pharmacological combination approaches may further define the mechanism and therapeutic potential of this regulatory axis.

## 5 Conclusion

Higher expression levels of CBX5 correlated with adverse clinicopathological features and shorter overall survival in the analyzed glioma datasets, with a significant prognostic association noted specifically in the CGGA693 cohort. In MGMT‐negative GBM models, the depletion of CBX5 enhanced the response to TMZ, increased apoptosis and sustained DNA damage, and restricted xenograft growth during TMZ treatment; conversely, CBX5 overexpression produced opposing effects in vitro. The RNA‐regulatory evidence aligned with METTL3‐dependent m^6^A modification and IGF2BP2‐associated transcript stabilization, which contributed to the maintenance of CBX5 expression. Furthermore, the partial rescue achieved through CBX5 re‐expression positioned CBX5 as a functional mediator of METTL3‐dependent alterations in cell survival and DNA damage during TMZ exposure. Collectively, these findings support a regulatory model wherein METTL3‐dependent m^6^A modification and IGF2BP2‐associated stabilization sustain CBX5 expression, thereby contributing to the TMZ‐resistant phenotype of MGMT‐negative GBM; this model necessitates further investigation in preclinical systems.

## Author Contributions

H.D. and R.C. were major contributors to the molecular experiments and manuscript writing. D.Q. and C.Q. performed statistical analysis of the experimental data. J.S. was responsible for the design and supervision of the experiments. All authors read and approved the final manuscript.

## Funding

This work was supported by Changzhou Municipal Science and Technology Bureau, CJ20245009.

## Ethics Statement

The study involving human tissue samples was approved by the Ethics Committee of The First People's Hospital of Changzhou (The Third Affiliated Hospital of Soochow University) (Approval No. (2026) Jiao 027).

## Consent

Written informed consent was obtained from all participants included in the study. All animal procedures were approved by the Institutional Animal Care and Use Committee of Shanghai Chengxi Biotechnology Co. Ltd. (Approval No. CX052603147).

## Conflicts of Interest

The authors declare no conflicts of interest.

## Supporting information


**Figure S1:** CBX5 overexpression promotes cell survival and attenuates apoptosis‐associated protein changes during TMZ treatment.
**Figure S2:** Supporting bioinformatic and expression analyses of the association between METTL3 and CBX5.

**Table S1:** Primer sequences used in this study.
**Table S2:** Primary antibodies used for Western blotting and selected reagents used in this study.

## Data Availability

The data that support the findings of this study are available from the corresponding author upon reasonable request.
