# Supplementary material for: METTL3 Increases Temozolomide Resistance in MGMT‐Negative Glioblastoma by Enhancing CBX5 mRNA Stability in an m6A‐IGF2BP2‐Dependent Manner
Source: CNS Neurosci Ther. 2026 Oct 1;32(10):e71164. doi: 10.1002/cns.71164 (PMC13628293; doi:10.1002/cns.71164)
Supplement: Supplementary file 1 [file CNS-32-e71164-s001.docx]

**Supplementary Tables**

**Table S1.** Primer sequences used in this study.

| Gene name | Species | Forward primer (5′–3′) | Reverse primer (5′–3′) |
| --- | --- | --- | --- |
| CBX5 | Human | GGCGCGTGGTTAAGGGACAA | CCTCTCTCAAAGCCCCGAGC |
| METTL3 | Human | CAAGCTGCACTTCAGACGAA | GCTTGGCGTGTGGTCTTT |
| APNG | Human | AAGGGCCACCTTACCCGACT | GAGTGGGCGGCTTCATCCTC |
| MGMT | Human | CACTGGACAGCCCTTTGGGG | ATAGCCTCGGGCTGGTGGAA |
| MLH1 | Human | GTTATTCGGCGGCTGGACGA | GGCCAAAGCCTCACCTCGAA |
| MSH2 | Human | AGACGCTGCAGTTGGAGAGC | GCCGTGCGCCGTATAGAAGT |
| MSH6 | Human | GAGATCGGTAGCGCCTGCTG | GCCCCTTGTTGGGCTGTCAT |
| FEN1 | Human | ACCGAGGACATGGACTGCCT | TCAAGTCGCCGCACGATCTC |
| XPC | Human | AGACTGTGTGCACGGTGTGG | GTGGGCAAAGGCTGGTCCAT |
| XRCC3 | Human | CGCCTGGTGGTCATCGACTC | ACCAGGAGCTGGTTAGCCCA |
| IGF2BP1 | Human | TTGGGACGCCATCAGTACCTA | TTGGCTAAACTCTCTACGACTCT |
| IGF2BP2 | Human | TGCACATCCCCAACTGTGAC | TGTAGAAGAGATGACACTCGGG |
| IGF2BP3 | Human | AGACACACTGAATCACCTGAAGT | AGGGCGACACTGCTTTTTCTT |
| YTHDF1 | Human | GGGGACAAGTGGGTCTCAAG | AGGGTGTCGCTGTGAAAGC |
| YTHDF2 | Human | GTTGGTAGCGGGTCCATTACT | GGTCTTCAGTTTAGGTTGCTGT |
| YTHDF3 | Human | GGTGTATTTAGTCAACCTGGGG | AAGAGAACTAGGTGGATAGCCAT |
| GAPDH | Human | GCGGGGCTCTCCAGAACATC | TCCACCACTGACACGTTGGC |

**Table S2.** Primary antibodies used for Western blotting and selected reagents used in this study.

| **Primary antibody** | **Catalog No.** | **Supplier** |
| --- | --- | --- |
| CBX5 | 11831-1-AP | Proteintech |
| MGMT | ab108630 | Abcam plc |
| Caspase-3 | 14220S | Cell Signaling Technology |
| Cleaved Caspase-3 | 9661S | Cell Signaling Technology |
| BCL-2 | 12789-1-AP | Proteintech |
| BAX | 50599-2-Ig | Proteintech |
| γH2AX | 9718S | Cell Signaling Technology |
| METTL3 | ab195352 | Abcam plc |
| FLAG | 14793S | Cell Signaling Technology |
| p-ATM | 5883T | Cell Signaling Technology |
| ATM | 2873T | Cell Signaling Technology |
| RAD51 | 8875T | Cell Signaling Technology |
| 53BP1 | 4937S | Cell Signaling Technology |
| p-DNA-PKcs | 68716T | Cell Signaling Technology |
| DNA-PKcs | 38168T | Cell Signaling Technology |
| IGF2BP2 | 14672T | Cell Signaling Technology |
| GAPDH | KC-5G5 | Aksomics |
| **Reagent** | **Catalog No.** | **Supplier** |
| Temozolomide | HY-17364 | MedChemExpress |
| Lipo3000 | L3000015 | Thermo Fisher Scientific (Invitrogen) |
